# Expression of SARS-CoV-2-related Receptors in Cells of the Neurovascular Unit: Implications for HIV-1 Infection

**DOI:** 10.21203/rs.3.rs-228960/v1

**Published:** 2021-02-24

**Authors:** Silvia Torices, Rosalba Cabrera, Michael Stangis, Oandy Naranjo, Daniel Adesse, Michal Toborek

**Affiliations:** University of Miami Miller School of Medicine: University of Miami School of Medicine; University of Miami Miller School of Medicine: University of Miami School of Medicine; University of Miami Miller School of Medicine: University of Miami School of Medicine; University of Miami Miller School of Medicine: University of Miami School of Medicine; FIOCRUZ: Fundacao Oswaldo Cruz; University of Miami School of Medicine

**Keywords:** ACE2, blood brain barrier, COVID-19, HIV-1, SARS-CoV-2, neuroinfection, TMPRSS2

## Abstract

**Background.:**

Neurological complications are common in patients affected by COVID-19 due to the ability of SARS-CoV-2 to infect brains. While the mechanisms of this process are not fully understood, it has been proposed that SARS-CoV-2 can infect the cells of the neurovascular units (NVU), which form the blood-brain barrier (BBB). The aim of the current study was to analyze the expression pattern of the main SARS-CoV-2 receptors in naïve and HIV-1-infected cells of the NVU in order to elucidate a possible pathway of the virus entry into the brain and a potential modulatory impact of HIV-1 in this process.

**Methods.:**

The gene and protein expression profile of ACE2, TMPRSS2, ADAM17, BSG, DPP4, AGTR2, ANPEP, cathepsin B and cathepsin L was assessed by qPCR and immunoblotting, respectively. In addition, we investigated if brain endothelial cells can be affected by the exposure to the S1 subunit of the S protein, the domain responsible for the direct binding of SARS-CoV-2 to the ACE2 receptors.

**Results.:**

The receptors involved in SARS-CoV-2 infection are coexpressed in the cells of the NVU, especially in astrocytes and microglial cells. These receptors are functionally active as exposure of endothelial cells to the SARS CoV-2 S1 protein subunit altered the expression pattern of tight junction proteins, such as claudin-5 and ZO-1. Additionally, HIV-1 infection upregulated ACE2 and TMPRSS2 expression in brain astrocytes and microglia cells.

**Conclusions.:**

These findings provide key insight into SARS-CoV-2 recognition by cells of the NVU and may help to develop possible treatment of CNS complications of COVID-19.

## Introduction

Coronavirus Disease-19 (COVID-19), which is caused by Severe Acute Respiratory Syndrome Corona Virus 2 (SARS-CoV-2) was first reported in Wuhan, China in December 2019. COVID-19 has become a pandemic, resulting in devastating morbidity and mortality worldwide due to lethal pneumonia and respiratory distress (1). In addition to systemic, respiratory, and cardiovascular complications (2), it has been reported that patients with COVID-19 can manifest symptoms of the central nervous system (CNS) infection, such as impaired consciousness, headache, encephalopathy, delirium, paresthesia, ataxia, encephalitis, as well as acute cerebrovascular events, such as ischemic or hemorrhagic stroke (3–12). COVID-19 patients also experience rhabdomyolysis (13), myositis (14) or peripheral nervous system symptoms, including Guillain-Barré syndrome (15), and taste or olfactory impairment (16, 17), The incidence of neurological complications is estimated to be around 37% in SARS-CoV-2-infected patients (5).

The SARS-CoV-2 genomic sequence shows a similarity of 75.5% with SARS-CoV-1 (18). Similar to SARS-CoV-1, SARS-CoV-2 binds to the host cell through the transmembrane protein angiotensin I converting enzyme 2 (ACE2) receptor using the spike protein (S protein). This protein is encoded by the S gene and is formed by the subunits S1 and S2. SARS-CoV-2 attaches to the host cells through the S1 subunit to ACE2 (19). Once attached, the host infection requires the expression of transmembrane serine protease 2 (TMPRSS2) for priming of the spike protein and viral entry into the cell (20–22). Along with ACE2 and TMPRSS2, several other molecules have been suggested to participate in SARS-CoV-2 entry into human cells, such as ADAM metallopeptidase domain 17 (ADAM17) (23, 24), dipeptidyl peptidase 4 (DPP4) (25, 26), angiotensin II receptor type 2 (AGTR2) (27, 28), basigin (BSG, also called extracellular matrix metalloproteinase inducer [EMMPRIN] or cluster of differentiation 147 [CD147]) (29, 30), aminopeptidase N (ANPEP) (31) and cathepsin B/L (32, 33).

The Central Nervous System (CNS) is well documented to be a target of beta coronaviruses infections such as SARS-CoV-1 (34–37) and SARS-CoV-2. Several studies detected SARS-CoV-2 in the brain and the cerebrospinal fluid of COVID-19 patients (5, 38–40). Some reports sustain that this neuroinvasion is due to retrograde axonal transport of the virus via the olfactory sensory neurons (3, 41, 42). In support of this notion, expression of ACE2 was demonstrated in human olfactory epithelium (17, 43) and olfactory dysfunction is a common symptom in SARS-CoV-2-infected individuals (5, 44, 45). Moreover, it was suggested that SARS-CoV-2 may reach the cerebral vasculature through the systemic circulatory system (25, 42) and crossing the blood brain barrier (BBB). While the mechanisms of this process are not fully understood, it has been suggested that it may be executed by infection of cells that compose the microvessels forming the BBB (46). The BBB, which is mainly formed by endothelial cells (EC), represents a barrier interface between the systemic circulation and the CNS. Surrounding the microvessels and coordinating function with EC are pericytes, astrocytes, neurons, and microglia forming functional elements of the BBB called the neurovascular units (NVU) (47). The barrier properties of the BBB are generated by high-resistance interendothelial tight junctions (TJs) formed by transmembrane proteins, such as claudin-5, and cytoplasmic proteins, such as zonula occludens (ZO-1), that limit the paracellular permeability between endothelial cells (48).

Older individuals and people with preexisting medical conditions are at higher risk of COVID-19 complications. These factors may be of particular significance in HIV-1 infection as HIV-1-infected patients experience accelerated aging and may suffer from immunodeficiency. In addition, HIV-1 is known to enter the CNS by altering the structures and properties of the BBB (49). In the brain, perivascular macrophages and microglia represent the primary cells infected with HIV (50, 51). Several investigations have also shown the capacity of astrocytes to be infected by HIV-1 (52–54) and recent evidence has emerged on the productive HIV-1 infection of brain pericytes (55–59). In contrast, no productive HIV-1 infection in EC has been reported (60).

In the present study, we aimed to analyze the expression profile of the main SARS-CoV-2 receptors in host cells forming the NVU in order to elucidate a possible pathway of the virus entry into the brain. Identifying the NVU cells with the greatest potential to be directly infected by SARS-CoV-2 would allow us to better understand the mechanisms of neuroinvasion and viral pathogenesis of SARS-CoV-2 in the brain. Taking into consideration possible interactions between SARS-CoV-2 and HIV-1, we also evaluated the expression of ACE2 and TMPRSS2, i.e., the main SARS-CoV-2 receptors, in these cells after HIV-1 infection. The obtained results indicate that the receptors involved in SARS-CoV-2 infection are coexpressed in the cells of the NVU, especially in astrocytes and microglial cells. Exposure of endothelial cells to the SARS CoV-2 S1 protein subunit altered the expression of TJ proteins, such as claudin-5 and ZO-1, potentially providing a route of SARS-CoV-2 entry into the brain. Additionally, HIV-1 infection upregulated ACE2 and TMPRSS2 expression in brain astrocytes and microglia. Overall, these findings provide key insight into the SARS-CoV-2 recognition by cells of the NVU and may help to develop possible treatment of CNS complications of COVID-19 disease.

## Material And Methods

### Cells cultures

Primary human brain microvascular endothelial cells were obtained from Cell Systems (Kirkland, WA, USA, Cat #ACBRI 376) and cultured in medium supplemented with CultureBoost, 10% serum, 100 units/mL penicillin and 100 μg/mL streptomycin. For TJ protein measurements, cells were exposed to 15 nM of the SARS-CoV-2 S protein S1 subunit (RayBiotech, Peachtree Corners, GA, Cat # 230–01101) in serum-free media without added antibiotics. Primary human astrocytes (ScienCell, Carlsbad, CA, USA, Cat #1800) were cultured in astrocyte-specific growth medium (ScienCell, #1801) supplemented with 2% FBS, astrocyte growth supplement, 100 units/mL penicillin, and 100 μg/mL streptomycin. Primary human brain vascular pericytes (ScienCell, Cat# 1200) were maintained in pericyte-specific growth medium (ScienCell, Cat# 1201) supplemented with 2% FBS, pericyte growth supplement, 100 units/mL penicillin, and 100 μg/mL streptomycin. Immortalized human microglia cell line (hμglia C20) created by SV40/hTERT-mediated immortalization (61) was kindly provided by Dr. Jonathan Karn (Case Western Reserve University, Ohio, OH, USA). Hμglia C20 cells were cultured as earlier described (62) in BrainPhys medium (StemCell Technologies, Vancouver, BC, Canada, Cat# 05791) containing 1X N2 supplement-A (Thermo Fisher Scientific, Cat #17502–048), 1X penicillin streptomycin (Gibco, Cat #15140122), 100 μg/mL normocin (InvivoGen, San Diego, CA, USA, Cat #ant-nr-1), 25 mM L-Glutamine (Thermo Fisher Scientific, Cat#25030081), 1% FBS, and 1 μM dexamethasone (Sigma-Aldrich, St. Louis, MO, USA Cat #D4902). Human embryonic kidney (HEK)-293T cells (ATCC, Manassas, VA, USA, Cat# CRL-11268) were cultured in Dulbecco’s Modified Eagle Medium (DMEM) (Thermo Fisher Scientific, Carlsbad, CA, USA, Cat#11995–065) and supplemented with 10% FBS (ScienCell, Cat# 0500), 100 units/mL penicillin, and 100 μg/mL streptomycin (Thermo Fisher Scientific, Cat# 15140–122). All cell cultures were maintained in 5% CO2 at 37°C.

### HIV-1 production and quantification

The HIV-1 pNL4–3 plasmid was acquired from the NIH AIDS Reagent Program (Division of AIDS, NIAID, National Institutes of Health). Viral stocks were generated by transfecting 10^7^ HEK-293T cells with 30 μg HIV-1 pNL4–3 plasmid using Lipofectamine 2000 (Thermo Fisher Scientific, Cat# 11668–027). Next day, medium was shifted to Opti-Mem (Thermo Fisher Scientific, Cat# 11058–021). After 48h, supernatants were collected, ltered using 0.45 μm-pore size filter (Millipore Sigma, Massachusetts, MA, USA, cat# 430314), and concentrated using weight exclusion columns (Millipore Sigma, Cat# UFC905024). Viral stocks were stored in −80°C. Viral concentration was analyzed in cell culture media by quantifying p24 antigen by HIV-1 p24 Antigen ELISA 2.0 according to the manufacturer’s instructions (ZeptoMetrix, Buffalo, NY, USA, Cat# 0801008). Primary human brain pericytes, astrocytes and immortalized human microglial cells were infected by incubation with 60 ng/mL of HIV-1 p24 for 24h or 48h.

### Quantitative PCR (qPCR)

Total RNA was isolated from cell culture lysates using RNeasy mini kit (Qiagen, Germantown, MD, Cat # 74104) following the manufacturer’s instructions and quantified using the Nanodrop 2000 (Thermo Fisher Scientific). RT-PCR was performed with a total of 100–800 ng of RNA using the qScript XLT 1-Step RT-qPCR ToughMix Low ROX (Quantabio, Beverly, MA, USA, Cat #89236–676) reaction mix and the Applied Biosystems 7500 system (Applied Biosystems, Foster City, CA). TaqMan Gene Expression Assays and ACE2 primer: Hs01085333_m1; TMPRSS2 primer: Hs00237175_m1; ADAM17 primer: Hs01041915_m1; BSG primer: Hs00936295_m1; DPP4 primer: Hs00897386_m1; AGTR2 primer: Hs02621316_s1; ANPEP primer: Hs00174265_m1; CATHEPSIN L primer: Hs02803063_cn and CATHEPSIN B primer: Hs02148115_cn were used for gene amplification. HIV-1 gag was measured using the following primers and probe: HIV-1gag_F 5′-GACATAAGACAGGGACCAAAGG-3′; HIV-1gag_R 5′-CTGGGTTTGCATTTTGGACC-3′; HIV-1gag_Probe 5′-AACTCTAAGAGCCGAGCAAGCTTCAC-3′. Human GAPDH was calculated for sample normalization. Coveted PCR product specificity was determined using melting curve assessment and gene expression fluctuations were determined by the ΔΔCt method, with Ct as the cycle number at threshold.

### Immunoblotting

After washing with phosphate-buffered saline (PBS), cells were lysed with Radio Immuno Precipitation Assay (RIPA) buffer containing protease inhibitors (Santa Cruz Biotechnology, Germany, EU, Cat# sc-24948a). Protein concentration was assessed using BCA Protein Assay Kit (Thermo Fisher Scientific, Cat# 23223). Samples were loaded on sodium dodecyl sulfate (SDS) polyacrylamide 4–20% Mini-PROTEAN TGX Stain-Free Protein Gels (Bio-Rad Laboratories, Hercules, CA, USA Cat# 4568094) and electrotransferred to a nitrocellulose membrane using the Trans-Blot Turbo Transfer System (Bio-Rad Laboratories, Cat# 170–4159). Afterward, the membranes were block with 5% bovine serum album (BSA) in TBS (Sigma-Aldrich, Cat# A7906–500G) for 1h. The blots were incubated in 5% BSA in TBS, overnight at 4°C with the following primary antibodies: mouse anti-ACE2 (Abcam, United Kingdom, Cat# ab15348), mouse anti-ZO-1 (Thermo Fisher Scientific, Cat# 339100), rabbit anti-claudin-5 (Thermo Fisher Scientific, Cat#341600) and rabbit anti-TMPRSS2 (Thermo Fisher Scientific, Cat# PA5–14264). Membranes were imaged using the Licor CLX imaging system after incubation with the secondary antibody in 5% BSA-TBS for 1h at room temperature (1:20000, LI-COR, Lincoln, NE, USA, Cat# 926–32210, Cat# 926–68070, Cat# 926–32211, cat# 926–68071). Target protein levels were normalized to anti-GAPDH (1:20000, Novus Biologicals Cat# NB600–502FR or Cat# NB600–5021R) or rabbit polyclonal anti α-tubulin antibody (1:1000, Thermo Fisher Scientific, Cat# 11224–1-AP). Signal quantification was performed using Image Studio 4.0 software (LI-COR).

### Statistical Analysis

All statistical analysis between experimental groups and controls were performed with GraphPad Prism 6 (GraphPad Software, La Jolla, CA, USA). Two-tailed student’s t test or one-way ANOVA following by Turkey’s multiple comparisons test were used for the analysis and *p* < 0.05 was considered significant.

## Results

### Cells of the NVU differentially express receptors involved in SARS-CoV-2 infection.

We first sought to characterize the expression levels of the main molecules involved in SARS-CoV-2 entry into host cells. RNA and protein were extracted from primary human brain EC, astrocytes, pericytes and immortalized human microglial cells. Expression of ACE2, TMPRSS2, ADAM17, BSG, DPP4, AGTR2, ANPEP, cathepsin L, and cathepsin B were analyzed by qPCR and immunoblotting. ACE2 was significantly more expressed at mRNA and protein levels in astrocytes compared to EC, pericytes, and microglia. Microglial cells presented the second highest expression of mRNA levels of ACE2 but the lowest protein levels of this receptor. EC and pericytes exhibited the lowest expression of ACE2 mRNA levels but higher protein levels than microglial cells (Figs. 1A-B).

Among studied cells, microglial cells expressed the highest TMPRSS2 mRNA and protein levels, with astrocytes being the next highest TMPRSS2 mRNA and protein producing cells (Figs. 1C-D). In contrast, the lowest expression of TMPRSS2 mRNA levels were detected in EC, followed by pericytes (Fig. 1C). EC and pericytes also expressed low levels of TMPRSS2 at the protein levels (Fig. 1D).

The highest expression of ADAM17 mRNA was found in astrocytes, followed by microglial cells, and EC and pericytes, which expressed the same levels of ADAM17 mRNA (Fig. 1E). The highest BSG mRNA levels were detected in microglial cells, followed by pericytes, astrocytes, and EC (Fig. 1F). DPP4 mRNA levels were the highest in microglial cells, followed by EC and pericytes with the same DPP4 mRNA expression, and astrocytes that exhibited the lowest expression (Fig. 1G). Microglial cells expressed the most pronounced AGTR2 mRNA levels, followed by pericytes, astrocytes and EC (Fig. 1H). In case of ANPEP, the highest mRNA expression was observed in pericytes, followed by EC, microglial cells, and astrocytes (Fig. 1I). Cathepsin B mRNA presented the most prominent levels in astrocytes, followed by EC, and pericytes and microglial cells, which expressed this gene at the same level (Fig. 1J). Lastly, the highest expression of cathepsin L was found in pericytes, followed by microglial cells, astrocytes, and EC (Fig. 1K).

### Claudin-5 and ZO-1 expression levels are influenced by exposure to the S1 subunit in primary human brain EC

EC are essential cells forming the BBB (63). Given our previous results showing that ACE2 protein and mRNA levels are lower in EC and pericytes when compared to other NVU cells, we investigated if EC can be affected by exposure to the S1 subunit of the SARS-CoV-2 S protein, the domain responsible for direct binding to the ACE2 receptor (19). We analyzed TJ protein expression changes, as their alterations may provide transendothelial entry of SARS-CoV-2 into the brain.

Human EC were exposed to a single dose of 15 nM of the SARS-CoV-2 S1 subunit and the expression level of claudin-5 and ZO-1, two key TJ proteins, were analyzed by immunoblotting. Claudin-5 expression level was significantly lower when cells were exposed to the S1 subunit for 3h as compared with untreated cells. Interestingly, there was a significant upregulation in the levels of claudin-5 when compared with the controls after 48h and 72h, suggesting recovery processes (Fig. 2A). We also observed a downregulation in ZO-1 protein after 3h exposure, followed by a significant increase after 72h exposure as compared with the untreated cells (Fig. 2B).

### Cells of the NVU are susceptible to HIV-1 infection

HIV-1 has the ability to cross the BBB and infecting the brain. Therefore, we measured infection of the cells of the NVU by the assessment of HIV-1 gag RNA levels by qPCR. The analyses were performed 24h and 48h post infection with 60 ng/ml of HIV-1. EC were excluded from these analyses because no productive HIV-1 infection in EC has been reported (60). The results indicated that astrocytes, pericytes, and microglia are susceptible to HIV-1 infection, confirming literature reports (51, 55, 56, 58). In addition, HIV-1 Gag RNA levels were significantly higher 24h after infection when compared to 48h after infection in all three cell types (Fig. 3A-C).

### ACE2 and TMPRSS2 expression levels increase after HIV-1 infection in primary human brain astrocytes

Astrocytes are the most abundant cell type of the CNS (64). Although it has been reported that astrocytes do not express the classical CD4 receptor for HIV-1 entry (65), several groups have identified HIV infection in astrocytes *in vivo* (52, 54–56, 58, 66). While some early studies described a non-productive infection (67), other reports indicated productive HIV-1 replication in astrocytes at low levels (52, 68). We also observed HIV-1 gag RNA expression in astrocytes after HIV-1 infection (Fig. 3A). Using the same infection paradigm as in Fig. 3, primary human astrocytes were infected with 60 ng/ml of HIV-1 for 24h and 48h and the expression of ACE2 and TMPRSS2 was measured by qPCR and immunoblotting. ACE2 mRNA and protein levels increased in astrocytes as the result of HIV-1 infection. ACE2 mRNA levels were elevated at both 24h and 48h post infection (Fig. 4A), while ACE2 protein levels increased only 48h post infection (Fig. 4C). In addition, TMPRSS2 mRNA levels were increased at 48h post HIV infection; however, no changes were found at the protein level (Figs. 4B, D, respectively).

### No changes in ACE2 or TMPRSS2 expression are detected after HIV-1 infection in primary human brain pericytes

Brain pericytes express the main HIV-1 receptors, CD4, CCR5 and CXCR4 and can be efficiently infected by HIV-1 (55, 59). Therefore, we investigated if HIV-1 infection could lead to alterations of ACE2 or TMPRSS2 expression. Infection with 60 ng/ml of HIV-1 for 24h or 48h did not result in any changes in the expression of ACE2 or TMPRSS2 at the mRNA (Fig. 5A, B) or protein level (Fig. 5C, D) in pericytes.

### HIV-1 infection of human microglial cells upregulates ACE2 and TMPRSS2 expression

Microglia are the main cell type supporting productive HIV-1 infection and generating a HIV-1 reservoir in the brain (50, 69–72). After infection with HIV-1, mRNA and protein expression of ACE2 and TMPRSS2 was evaluated by qPCR and immunoblotting, respectively. HIV-1 infection resulted in a significant increase in ACE2 mRNA level at 24h but not at 48h as compared to mock-infected cells (Fig. 6A). However, no changes in ACE2 expression were detected at the protein level (Fig. 6C). The expression pattern of TMPRSS2 indicated a significant increase at the mRNA levels at both 24h and 48h post infection compared to mock-infected cells (Fig. 6B). In addition, a significant increase in TMPRSS2 protein level was observed 24h post HIV-1 infection (Fig. 6D).

## Discussion

Neurological complications are frequent in patients affected by COVID-19 (4, 5). Several studies have reported that SARS-CoV-2 can invade the CNS; however, the mechanisms of this process remain unclear. It was proposed that an invasion of the CNS by infection of the BBB cells may be responsible for this effect (11, 40, 73). SARS-CoV-2 entry into the cells uses the ACE2 receptor and the serine protease TMPRSS2 to allow the interaction with the viral spike protein S, followed by a membrane fusion resulting in cell viral entry (20). In addition, a variety of other molecules have been suggested to be involved in the SARS-CoV-2 internalization into the host cell. Some of them are ADAM17 (23), DPP4 (25), AGTR2 (27), BSG (29), ANPEP (31) and Cathepsin B/L (32).

Specific cells of the NVU that can be implicated in SARS-CoV-2 neuroinvasion have not been identified. In particular, a comprehensive pro le of the host receptors on the NVU cells that can be involved in SARS-CoV-2 entry into the brain is unknown. Such studies are important, because they can identify which of the NVU cell types may provide the main route of SARS-CoV-2 entry into the CNS. Previous studies indicated that astrocytes, microglial cells, and endothelial cells express ACE2 (30) (74, 75). Recently, high expression levels of ACE2 in adult human heart pericytes (76, 77) and mouse olfactory bulb pericytes (78) have been reported. However, brain pericytes have different origin and no studies have yet described expression of ACE2 in human BBB pericytes. Of the NVU cells, expression of TMPRSS2 has only been studied and reported in microglial cells (30). Studies have shown a low level of expression of TMPRSS2 in blood vessel endothelial cells; however, there are no reports of TMPRSS2 expression in microvascular endothelial cells that compose the BBB (79).

The current study describes the pro le of expression of the main receptors involved in SARS-CoV-2 infection and entry into the NVU cells. At the mRNA levels, our results indicated that astrocytes displayed the highest expression of ACE2, ADAM17 and cathepsin B as well as the second highest levels of TMPRSS2. They also expressed mRNA for BSG, AGTR2, and cathepsin L but the lowest mRNA expression for DPP4 and ANPEP. Among the cells of the NVU, the second highest mRNA levels for ACE2 were detected in microglial cells; however, their ACE2 protein level expression was relatively low. Microglial cells also exhibited the highest levels of TMPRSS2, BSG, DPP4 and AGTR2 mRNA. mRNA levels for ADAM17, ANPEP and cathepsin B and L were also prominently expressed in these cells. These results support literature reports showing ACE2 and TMPRSS2 mRNA and protein levels in human brain microglial cells (30). Microglia were also reported to express ADAM17 (80) and cathepsin B and L (81).

EC and pericytes expressed ACE2 and TMPRSS2 mRNA at low levels as compared to astrocytes and microglial cells. Similar to TMPRSS2 mRNA, BSG, AGTR2, and cathepsin L mRNA levels in EC were the lowest among studied cells of the NVU. These findings are important because EC generate the main interface between the blood stream and the brain. Thus, a low expression of ACE2 and TMPRSS2 may provide some protection against SARS-CoV-2 entry into the brain. On the other hand, protein expression of ACE2 and TMPRSS2 in EC was higher and comparable to other cells of the NVU. We next analyzed if ACE2 expression on EC can induce phenotype changes upon exposure to the S1 subunit of the SARS CoV-2 S protein, the domain responsible for direct binding to the ACE2 receptor (19). EC treatment with the S1 subunit resulted in TJ dual-stage response pattern, where claudin-5 and ZO-1 expression levels decreased 3h after exposure, followed by an increase after 48h and 72h as compared with the controls (Fig. 2). Disruption of TJ protein expression in EC exposed to the S1 subunit is consistent with observations that the S protein alters barrier function in a model of human blood-brain barrier (82).

Within the NVU, EC closely interact with pericytes. Indeed, pericytes wrap around the brain endothelium via cytoplasmic processes that extend along the abluminal surface of the endothelium and cover close to 100% of the brain microvascular endothelium. Part of the pericyte-endothelial interface is separated by the basement membrane; however, pericytes also remain in direct contact with endothelial cells via the peg-socket type of arrangement (57, 83). Therefore, it was important that mRNA expression of ACE2 and TMPRSS2 was also low in pericytes.

Several studies have focused on investigating a possible association between ACE2 expression and the interferon (IFN)-signaling pathway. It was reported that administration of exogenous IFN-γ downregulated the expression of ACE2 receptor in interferon-deficient Vero E6 cells (84). Interestingly, recent reports suggested ACE2 to be one of the interferon-stimulated genes (ISG) (20, 85, 86). Indeed, it was shown that type I IFNs, and to a lesser extent type II IFNs, can significantly upregulate ACE2 expression levels in human nasal epithelial cells. In addition, type I IFNs can upregulate ACE2 in other cells of the epithelial barrier tissue, such as primary bronchial cells and keratinocytes (85). The finding that ACE2 is an ISG has broad implications, including HIV infection. In fact, HIV-1 entry into host cells stimulates an IFN-driven induction of ISGs as part of the cellular antiviral defense network (87).

The CNS is susceptible to infection by lentiviruses, such as HIV-1, through the viral entry from the periphery into the brain (73, 88). Several studies have shown that HIV-1 infection modulates gene and protein expression in the host cells (55, 87). Therefore, we proposed to evaluate whether HIV-1 infection modulates the expression levels of ACE2 and TMPRSS2. We first examined the efficiency of HIV-1 infection of NVU cells. Primary human brain astrocytes, pericytes and human microglia were infected with HIV-1 for 24h and 48h and the expression levels of HIV-1 gag were measured by qPCR. In agreement with previous studies, we found a successful infection of HIV-1 in microglial cells (50, 51), pericytes (55–57), and astrocytes (52–54). There was significantly higher expression of HIV-1 gag 24h after infection compared to 48h in microglia, pericytes, and to a lesser degree in astrocytes (Fig. 3).

Next, we evaluated if ACE2 and/or TMPRSS2 levels are affected by HIV-1 infection. Overall, a significant increase in ACE2 and TMPRSS2 at both mRNA and protein levels were observed in HIV-1 infected astrocytes and, especially, in microglial cells (Figs. 4 and 6). These effects may be related to IFN-α/β signaling that was reported to regulate HIV infection in both microglia and astrocytes (89, 90). Indeed, astrocytes and microglia are the main producers of IFN during in ammatory response in the CNS (91). Microglia are also the cell type that is most susceptible to HIV-1 infection within the CNS.

In contrast, no changes in ACE2 or TMPRSS2 mRNA or proteins were detected in pericytes upon HIV infection (Fig. 5), even though pericytes can be productively infected by HIV-1 and respond to in ammatory signals (92–94). On the other hand, HIV-1 infection in pericytes appears to not be influenced by IFNs as interferon-α, -β, and -γ levels were not affected in HIV-infected pericytes (56). These results may confirm the notion that ACE2 expression is regulated by IFNs upon HIV-1 infection. In support of this notion, an increase in ACE2 expression in secretory cells of the nasal epithelium has been reported in infection by influenza virus (85). The influenza virus is recognized to be an efficient inducer of the IFN pathway similar to HIV-1 (95). An overexpression of ACE2 mRNA in CD4 + T cells has also been described in patients with systemic lupus erythematosus (96), a disease that is associated with interferon induction (97, 98).

Elevated COVID-19 mortality in patients with immunocompromised immune systems (99) suggested that people with HIV-1 might be of an increased risk of COVID-19-related complications and death. Surprisingly, several studies indicated that COVID-19 pathology does not markedly differ between HIV-1-infected individuals and the general population (100–104). These findings can be explained as the result of successful antiretroviral therapy (ART) that decrease plasma HIV-1 viral load to undetectable levels (105–108). On the other hand, ART is less efficient in treatment of HIV-1 infection in the brain due to the barrier function of the BBB, which limits brain penetration of antiretroviral drugs. Thus, the interactions between HIV-1 and COVID-19 in the CNS remain a threat. In addition, HIV-1-infected patients who are not on ART might be at increased risk of SARS-CoV-2 infection and more severe COVID-19 outcomes.

In conclusion, the present study describes the coexpression of the main receptors involved in SARS-CoV-2 infection in the cells of the NVU, suggesting their susceptibility to SARS-CoV-2 infection. Among NVU cells, the most prominent expression of SARS-CoV-2 receptors was observed in astrocytes and microglial cells. Additionally, HIV-1 infection of brain astrocytes and microglia cells upregulated ACE2 and TMPRSS2 expression levels. These findings will help to better understand the pathology of CNS infection by SARS-CoV-2 and the role of HIV-1 infection in the progression of COVID-19.

## Data Availability

All data generated during and/or analyzed during the current study are included in this published article. All source data supporting the findings of this manuscript are available from the corresponding authors upon request.

## Abbreviations

ACE2: angiotensin I converting enzyme 2
ADAM17: a disintegrin and metalloproteinase 17
AGTR2: angiotensin II receptor type 2
ANPEP: aminopeptidase N
BBB: blood-brain barrier
BCA: bicinchoninic acid
BSG: basigin
CNS: central nervous system
COVID-19: coronavirus disease-19
DPP4: dipeptidyl peptidase 4
EC: endothelial cells
FBS: fetal bovine serum
GAPDH: glyceraldehyde 3-phosphate dehydrogenase
HEK: human embryonic kidney
HIV-1: human immunodeficiency virus
IFN: interferon
ISG: interferon-stimulated genes
NVU: neurovascular units
PBS: phosphate-buffered saline
qPCR: quantitative PCR
S protein: spike protein
S1: S1 subunit of the Spike protein
SARS-CoV-2: severe acute respiratory syndrome-coronavirus 2
TMPRSS2: transmembrane protease, serine 2
ZO-1: zonula occludens-1
TJ: tight junction
